# Influence of Overweight and Obesity on Morbidity and Mortality among Hospitalized Patients in Sri Lanka: A Single-Center Analysis

**DOI:** 10.1155/2022/9172365

**Published:** 2022-08-18

**Authors:** M. D. S. A. Dilrukshi, V. Thotamuna, D. J. Senarath Yapa, L. De Silva, P. Ranasinghe, P. Katulanda

**Affiliations:** ^1^National Hospital of Sri Lanka, Colombo, Sri Lanka; ^2^Diabetes Trial Unit, Faculty of Medicine, University of Colombo, Colombo, Sri Lanka; ^3^Department of Pharmacology, Faculty of Medicine, University of Colombo, Colombo, Sri Lanka; ^4^Department of Clinical Medicine, Faculty of Medicine, University of Colombo, Colombo, Sri Lanka

## Abstract

**Background:**

Current evidence regarding the association between overweight and obesity and in-hospital morbidity and mortality is inconsistent and South Asian populations are underrepresented.

**Methods:**

Data relevant to anthropometry, hospital outcomes, complications, and medical diagnoses of all acute medical admissions to the National Hospital of Sri Lanka were collected over a period of 3 months. Analysis was performed with WHO international (ICs) and Asian obesity cut-offs (ACs).

**Results:**

Sample size was 2,128 (median age: 57 years [IQR: 42, 67], males: 49.7%). High prevalence of overweight (23.5%), generalized obesity (10.4%), central obesity (28.5%), and underweight (15.4%) was observed (ICs). Patients with either generalized or central obesity had significantly higher in-hospital mortality (4.8% versus 2.5%, *p* = 0.031) and acute kidney injury (AKI) (3.9% versus 1.2%) (*p* = 0.001) compared to normal weight. With ACs, overweight and obesity prevalence increased, without any significant increment in morbidity and mortality, but median length of hospital stay was significantly reduced in patients with generalized obesity compared to normal (3 [IQR: 2, 5] versus 4 [IQR: 2, 6], *p* = 0.014). Infections (44.4%) and cardiovascular diseases (CVDs) (25.9%) were the most common causes of admission. Overweight and generalized obesity or central obesity were associated with increased prevalence of acute CVDs and CVD risk factors and lower prevalence of acute infections, whilst underweight showed an inverse association.

**Conclusion:**

A double burden of malnutrition and diseases were noted among hospital admissions, with obesity being a risk factor for in-hospital all-cause mortality and AKI. Overweight and obesity were associated with increased CVDs and reduced infections. Larger prospective studies are required to characterize these associations among South Asians.

## 1. Introduction

Obesity is characterized by excessive or abnormal accumulation of body fat leading to adverse health outcomes. Different morbidities associated with overweight or obesity include diseases with increased cardiovascular disease (CVD) risk (e.g., type-2 diabetes mellitus [T2DM] and dyslipidemia), functional difficulties (e.g., osteoarthritis [OA]), and psychological disturbances [1], whilst CVDs and cancers appear to drive the excess mortality [2]. Given the above, it can be expected that overweight and obesity could adversely affect hospital outcomes, morbidity, and mortality. However, existing evidence is inconsistent. For example, outcomes of a regional database [3] of patients undergoing coronary artery bypass grafting (CABG) (*n* = 13,637) and a large nationwide hospital survey (*n* = 800, 417; 74.5% with body mass index (BMI) >30 kg/m^2^) [4] showed that overweight, obesity, and morbid obesity (BMI >40 kg/m^2^) are associated with increased risk of in-hospital mortality and morbidity. However, a multinational survey (*n* = 97,344) of non-critically ill adult hospital admissions [5] and a prospective acute coronary syndrome (ACS) registry (*n* = 4,044) [6] data reported an inverse relationship between in-hospital mortality and generalized obesity and a trend towards reduced mortality with central obesity.

BMI is a measure of nutritional status and the most widely used practical tool to classify obesity. Increased BMI correlates with excessive body fat percentage and consistently associated with increased mortality and morbidity. WC is a measure of abdominal or central adiposity, correlating well with BMI and with mortality and morbidity (8). It is at times used as an alternative to BMI [7]. The World Health Organization (WHO) recommends optimal cut-offs for BMI and WC (Supplementary File 1: Tables 1 and 2). However, given the high prevalence of T2DM and other CVD risk factors occurring among Asians whose average BMI is below international cut-off (IC) point of 25 mg·Kg^2^, lower cut-offs are recommended for Asians [8]. Furthermore, prevalence of overweight and obesity varies significantly among Caucasians and Asians likely due to different dynamics in diet, genetics, lifestyles, and socioeconomic factors: the prevalence of overweight and that of obesity in USA were ≥30% and ≥20%, respectively, in 2017 [9], whereas these figures were 16.8% and 3.7%, respectively, in Sri Lanka in 2010 with same BMI cut-offs [10]. It is noteworthy that clear mapping of overweight and obesity prevalence in South Asia is limited by unavailability of nationwide studies and differences in cut-offs used [11]. Furthermore, almost all the above published studies were from Europe or USA with predominant patients of Caucasian origin. Despite the significant aforementioned differences, no reliable hospital outcomes studies in relation to obesity are available in Asian populations. Thus, we set out to evaluate the burden of overweight and obesity among hospitalized patients in a tertiary care setting in Sri Lanka (SL), a developing South Asian country, and to study its association with in-hospital outcomes, complications, and morbidity.

## 2. Methods

### 2.1. Study Design, Setting, and Participants

This retrospective single-center study was carried out in the National Hospital of Sri Lanka (NHSL), Colombo, the largest tertiary care hospital in the country with a 3,000-bed capacity and 8 general medical units (GMUs). All the adult patients newly admitted to the 8 GMUs with an acute medical illness from September to November 2019 were included by consecutive sampling. Patients without an acute illness admitted for routine investigations or treatment and pregnant or postpartum women were excluded. Informed written consent was obtained from study participants or their legal guardians prior to enrolment.

### 2.2. Data Collection, Measurements, and Definitions

Data relevant to the study [demographic data, height, and weight or WC, intensive care unit (ICU) admissions, ICU or hospital length of stay, diagnoses of the acute medical illnesses, chronic diseases related to obesity: ischemic heart disease (IHD), heart failure (HF), T2DM, dyslipidemia, hypertension, OA, Obstructive Sleep Apnea (OSA), and Venous Thrombosis (VT), and in-hospital complications: pressure ulcers, hospital-acquired infections (HAI), and acute kidney injury (AKI)] were collected by the perusal of patient's clinical records (PCRs) and this was carried out using a preformatted questionnaire (Supplementary File 2) by two trained research assistants under the supervision of the principal investigator (SADMD). GMUs were numbered from 1 to 8 and four units were allocated to each research assistant who visited these wards every day including weekends twice per day during the working hours. Clinical diagnoses (for acute medical illnesses and complications) made by the consultant physicians of the relevant GMUs were collected upon discharge as recorded in PCRs. Details of chronic medical conditions were obtained from PCRs and double checked by interviewing patients and going through their clinic records. Acute and chronic medical conditions were subsequently coded and converted into composite variables using the International Classification of Diseases, 10th Revision, Clinical Modification.

Weight and height data were obtained as recorded in PCRs which were measured by medical ward staff following standard protocol and BMI was calculated (kg/m^2^). When these data were not available, WC was measured midway between the iliac crest and lower rib margin at the end of normal expiration using a plastic flexible tape to the nearest 0.1 cm by trained research assistants. These research assistants were trained prior to data collection to minimize intra- and interobserver variances. BMI and WC were classified during analysis as per ICs and ACs (Supplementary File 1: Tables 1 and 2). Additionally, a separate variable was created (“generalized or central obesity”) by combining the generalized and central obesity groups and was done separately using both ICs and ACs.

### 2.3. Statistical Analysis

Descriptive statistics were used to examine the prevalence and the difference in the distribution of acute and chronic medical conditions according to BMI and WC categories; the significance of the differences between proportions (%) and medians was tested using chi-square for categorical variables and Mann-Whitney *U* test for continuous variables. Composite variables were created for acute diseases as above (Supplementary File 1: Table 3). Age distribution curves were plotted for different BMI and WC groups. A backward stepwise binary logistic regression analysis was performed to determine the association between BMI and WC categories and all-cause in-hospital mortality, adjusting for age, gender, and acute or chronic conditions which showed significant associations during univariate analysis. In all statistical analyses, *p* values <0.05 were considered significant. Data were analyzed using IBM SPSS Statistics for Windows, Version 22.0 (IBM Corp., Armonk, NY, USA) statistical software package.

## 3. Results

### 3.1. Sample Characteristics

**Figure 1:**
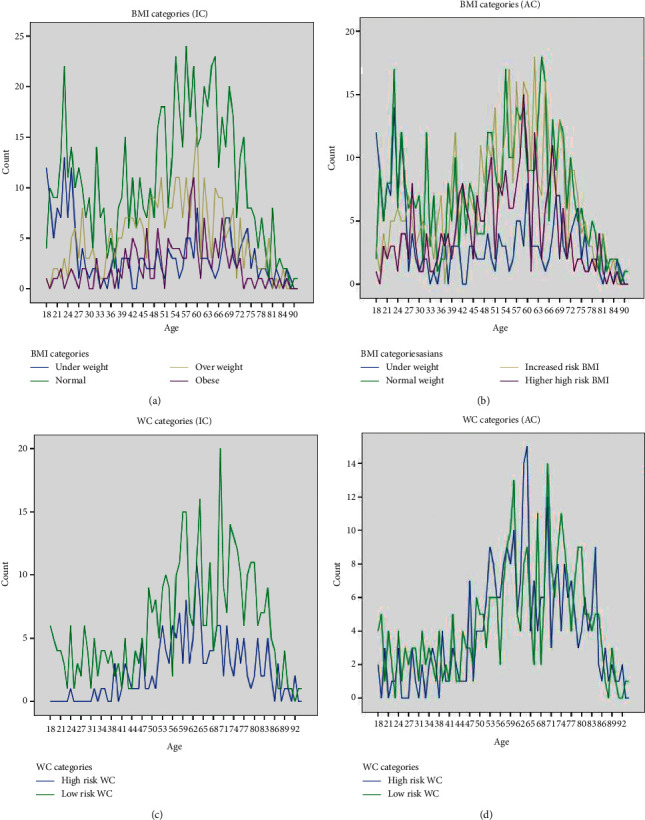
Distribution of BMI (kg/m^2^) and WC (cm), according to age according to the international cut-offs and Asian cut-offs in (a), (b), (c), and (d), respectively.

The sample included 2,128 patients [median age: 54 years (interquartile range (IQR): 42, 67), males: 49.7%]. Of the total, 1,500 and 628 patients had BMI and WC measured, respectively, with none having both measurements. BMI distribution was right-skewed [median: 23 kg/m^2^ (IQR: 20, 26)] and WC distribution was bimodal [median: 8 cm (IQR: 68, 92)] (Supplementary File 1: Figure 1). Generalized and central obesity were predominantly seen among middle age groups (Figure 1). Females had a significantly higher BMI or WC when compared with males (Table 1).

### 3.2. Prevalence of Overweight and Obesity

**Figure 2:**
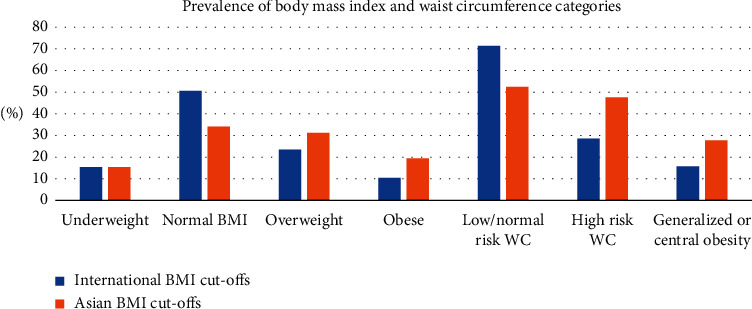
Prevalence of BMI and WC categories in the cohort.

According to ICs, the prevalence of overweight, generalized obesity, and central obesity was 23.5% (males: 48.6%, *p* = 0.253), 10.4% (males: 33.3%, *p* < 0.0001), and 28.5% (males: 13.4%, *p* < 0.0001), respectively (Table 1). Overall, 15.7% had either generalized or central obesity with a female preponderance (77.3%, *p* < 0.0001). With ACs, the prevalence of overweight, generalized obesity, and central obesity increased up to 31.1% (males: 53.5%, *p* = 0.257), 19.3% (males: 36.6%, *p* < 0.0001), and 47.6% (males: 28.1%, *p* < 0.0001), respectively, and the prevalence of either generalized or central obesity increased to 27.7% (males: 32.3%, *p* < 0.0001). Of note, the prevalence of underweight was 15.4% in both cut-offs (males: 53.9%, *p* = 0.437) (Figure 2).

### 3.3. In-Hospital Outcomes

Of the total, 49 died with a crude mortality rate of 2.3% (95% CI: 1.7%–3.1%); males had slightly higher rate (2.6% [95% CI: 1.7%–3.1%]) than females (2.1% [95% CI: 1.3%–3.1%]) (*p* = 0.434). There was no significant difference in in-hospital all-cause mortality among BMI and WC categories when compared to normal in both cut-offs. However, the group with either generalized or central obesity (ICs) had a significantly higher mortality compared to patients with normal BMI or WC parameters (normal BMI or WC: 2.5% versus generalized or central obesity: 4.8%, *p* = 0.031) but not when classified with ACs (*p* = 0.226).

Eleven patients (0.5%) needed ICU care (Table 2). In ICs, patients with underweight had a higher ICU admission rate compared to normal weight (NW) (1.7% versus 0.1%, *p*=0.003) but there was no significant association between median length of stay in ICU and overweight or obesity categories. In ACs, no ICU admissions were reported in the NW group. Median length of stay in hospital for the total cohort [4 (IQR: 2, 6) days] did not significantly vary between BMI or WC groups in ICs but that was significantly reduced among patients with generalized obesity compared to NW (3 [IQR: 2, 5] versus 4 [IQR: 2, 6], *p*=0.014) with ACs. Of the total, 35 (1.6%) had AKIs, 39 (1.8%) had HAIs, and 7 (0.3%) had pressure ulcers. Patients with central obesity (6.1% versus 1.3%) (*p*=0.001) and “generalized or central obesity” (3.9% versus 1.2%) (*p*=0.001) had a higher risk of AKI when compared to normal with ICs and a trend towards higher risk with central obesity (4% versus 1.5%) (*p*=0.054) in ACs. No significant association was noted in other complications (in both classifications).

### 3.4. In-Hospital Morbidity (Acute Medical Conditions)

Acute infections were the leading cause (44.4%) of admissions followed by acute CVDs (25.9%) (Table 3). Male predominance in prevalence was noted for acute CVDs (*p* = 0.050), respiratory diseases (*p* = 0.030), and GI/liver diseases (*p* = 0.009), whilst a female predominance was seen for diabetes-related admissions (*p* = 0.036) (Supplementary File 1: Table 4). Prevalence patterns of BMI and WC categories among acute diseases are presented in Table 3. When ICs were used (Table 3 and Supplementary File 1: Tables 5.1–5.4), in comparison to NW, the prevalence of acute CVDs (22.6% of NW, 30.3% of low-risk WC, and 25.5% of normal BMI or WC) was notably increased among high BMI and WC groups: overweight (30.1%, *p* = 0.007), generalized obesity (30.1%, *p* = 0.040), central obesity (33.5%, *p* = 0.430), and “generalized or central obesity” (31.9%, *p* = 0.018) groups. An inverse prevalence pattern was noted with acute infections; the prevalence of acute infections was 49.1% in NW, 37% in low-risk WC, and 44.6% in normal BMI or WC, in contrast to 39.2% (*p* = 0.002) in overweight, 41.0% (*p* = 0.065) in generalized obesity, 35.8% (*p* = 0.775) in central obesity, and 38.2% (*p* = 0.036) in “generalized or central obesity” group. In ACs (Table 3 and Supplementary File 1: Tables 6.1–6.4), the prevalence of high-risk BMI and WC noticeably increased and the pattern of associations mirrored that of ICs. In comparison to NW (ICs), patients with underweight had higher prevalence of acute infections (59.1% versus 49.1%, *p* = 0.008) and acute respiratory diseases (8.3% versus 3.8%, *p* = 0.006) but significantly lower prevalence of CVDs (22.6% versus 13.9%, *p* = 0.004) (Supplementary File 1: Table 7.1). Similar results were obtained with ACs (Supplementary File 1: Table 7.2).

In subgroup analysis, prevalence of acute CVDs and infections was lower among males with higher BMI and WC compared to males with normal parameters, with an inverse association observed among females (in ICs or ACs) (Supplementary File 1: Tables 5 and 6). Additionally, females with underweight had higher prevalence of infections and CVDs, whilst males with underweight had lower prevalence of infections but higher prevalence of acute CVDs (in ICs or ACs) compared to the NW counterpart (Supplementary File 1: Tables 7.1 and 7.2).

### 3.5. In-Hospital Morbidity (Chronic Medical Conditions)

Prevalence of BMI and WC categories among chronic diseases is presented in Table 4. Overall, hypertension was the highest prevalent (39.9%) followed by T2DM (39.3%) and IHD (20.5%). Dyslipidemia (66.4%, *p* < 0.001), hypertension (56.1%, *p* < 0.001), T2DM (55.1%, *p* < 0.001), OA (87.8%, *p* < 0.001), and VT (91.6%, *p*=0.004) were significantly more prevalent among females. (Supplementary File 1: Table 8). In ICs (Table 4 and Supplementary File 1: Tables 9.1–9.4), patients with overweight (42.9%, *p* < 0.001), generalized obesity (59%, *p* < 0.001), and central obesity (65.7%, *p* < 0.0001) were at higher risk of hypertension in comparison to patients with NW (32.1%) and low-risk WC (43.2%). Further, patients with generalized obesity and central obesity had significantly higher risk of dyslipidemia [27.6% versus 17% (*p*=0.002) and 19.2% versus 11.4% (*p*=0.01)] and T2DM [49.4% versus 37.4% (*p*=0.005) and 62.4% versus 39.4% (*p* < 0.0001)], compared to patients with NW and low-risk WC. Significantly high prevalence of IHD was noted among patients with overweight (23.6% versus 18.1%, *p*=0.033) and central obesity (29.1% versus 21.4%, *p*=0.041). OA prevalence was significantly higher among patients with overweight (2.8%, *p*=0.050) and generalized obesity (4.5%, *p*=0.004) compared to NW (1.2%). The group of patients with “generalized or central obesity” had higher risk of HTN (*p* < 0.0001), T2DM (*p* < 0.0001), IHD (*p*=0.001), and dyslipidemia (*p*=0.031).

In ACs (Table 4 and Supplementary File 1: Tables 10.1–10.4), the prevalence of chronic diseases was notably increased among patients with high BMI or WC. However, the prevalence of none of the chronic diseases was significantly associated with being overweight. A pattern similar to ICs was observed with obesity groups.

Of note, in ICs, patients with underweight had significantly lower prevalence of T2DM (16.1% versus 37.4%, *p* < 0.0001), dyslipidemia (7% versus 17% *p* < 0.0001), and hypertension (21.7% versus 32.1%, *p*=0.003) compared to NW and similar results were obtained with ACs.

In subgroup analysis, males with overweight, generalized obesity, and central obesity had noticeably lower prevalence of chronic diseases (except for IHD) compared to males with NW, whilst an inverse association was observed with females (Supplementary Tables 9 and 10). In contrast, males with underweight had significantly higher prevalence of T2DM and dyslipidemia, whilst females with underweight had significantly lower prevalence of T2DM, hypertension, and dyslipidemia (Supplementary Tables 11.1-11.2).

### 3.6. Results of Binary Logistic Regression Analysis

Binary logistic regression was performed to predict in-hospital mortality with age, acute renal disease, T2DM, hypertension, HF, and “either generalized or central obesity” as independent variables that showed significant association with in-hospital mortality in univariate analysis. However, the model could not accurately classify mortality events given the very low rate and hence could not be used to estimate the strength of the association.

## 4. Discussion

To the best of our knowledge, this single-center study to assess in-hospital outcomes in relation to overweight and obesity is the first from a South Asian country. We observed high prevalence of overweight, generalized obesity, and central obesity in the hospital cohort with equally high prevalence of underweight. Females were more likely to have higher BMI or WC and associated metabolic risk. Obesity appeared to be a significant risk factor for in-hospital all-cause mortality and it was associated with increased risk of AKI (in ICs) and reduced median LOS in hospital (in ACs). Additionally, acute infections and CVDs were the leading causes of hospital admissions (double burden of diseases) and higher BMI and WC were associated with increased prevalence of acute CVDs and CVD risk factors and reduced prevalence of infections, whilst patients with underweight showed an inverse prevalence pattern.

Of note, the prevalence of overweight in the cohort was significantly higher and the prevalence of generalized obesity and that of central obesity were more than two-fold compared to the general SL population; in ICs, the population prevalence of overweight, generalized obesity, and central obesity was 16.8%, 3.7%, and 10.8% (25.2%, 9.2%, and 26.2% in ACs), respectively, in 2010 [10]. As the published population data are nearly a decade old, this increased prevalence might reflect the increasing epidemic of overweight and obesity reported elsewhere in the world. A contemporary regional study from SL supports this notion; “the Colombo urban study” reported 37% prevalence of overweight, 15.8% of obesity in the Colombo district in 2019 (in ICs) [12]. Nevertheless, as obese patients are more likely to admit to medical services, ascertainment bias, as well as a small sample size, could have also contributed to the observed increased prevalence. We also noted a significant female preponderance in the prevalence of higher BMI or WC in accordance with existing literature and possible different metabolic risk profile compared to males in subgroup analysis. Plausible factors for enhanced risk in females are gender differences in socioeconomic and cultural factors, pregnancy-related feeding practices, and hormone-related factors: increased mineralocorticoid receptor activation, aberrant estrogen signaling, and elevated androgens among females [13].

Furthermore, obesity-associated increased in-hospital mortality risk in our cohort could be driven by increased prevalence of acute CVDs and CVD risk factors among these patients. We could not elaborate on the strength of this association given the very low in-hospital mortality rate, likely related to small sample size and the study being performed in the largest tertiary care center of the country with efficient and free healthcare delivery. Reported in-hospital mortality studies have several limitations; study samples predominantly included patients with either obesity and/or morbid obesity [4, 14] or specific patient groups (i.e., ACS, CABG, and critically-ill patients) [15, 16] or certain ethnic categories (predominantly Caucasians), comparing mortality risks in broad BMI groups (BMI <30 kg/m^2^ versus BMI >30 kg/m^2^) [2, 17, 18]; only few studies evaluated the association of central obesity [6], hence lack of generalizability, and this likely led to inconsistent mortality related outcomes.

Length of hospital or ICU stay and ICU admissions are common in-hospital outcome determinants reported in relation to obesity, and, quite consistently, obesity increased both parameters leading to increased healthcare related cost [3, 14, 16, 17, 19]. On the contrary, we observed reduced hospital length of stay with generalized obesity in ACs (no association with ICs) which is an interesting finding. However, it is noteworthy that characteristics of our cohort (patients with only South Asian origin were included with relatively low prevalence of overweight and obesity, including a broad spectrum of acute medical conditions, as well as infections being the commonest cause of admissions) are notably different from reported studies (predominantly including Caucasians with significantly higher prevalence of obesity or morbid obesity, conducted among patients with specific medical conditions (i.e., ACs), with CVDs being the most common cause of admissions) and hence are incomparable in this regard. With regard to in-hospital complications, obesity is found to be associated with increased risk of AKI among critically ill patients with several plausible mechanisms: altered renal hemodynamics-hyperfiltration syndrome, increased oxidative stress, altered or increased inflammatory mediator response for acute illness, suboptimal fluid or vasopressor prescriptions due to difficulties in assessment, and imprecise dosing of potentially nephrotoxic medications [20, 21]. However, we note that our data set is incomplete with complications, as there may be underreporting of complications and/or other diagnoses in the PCRs at discharge (more often, only the main diagnosis for the particular admission was recorded).

Moreover, we observed significantly lower prevalence of acute infections among patients with high BMI or WC compared to normal, and outcomes of existing literature on this regard are quite inconsistent; epidemiological studies report increased risk of infections in obesity that is postulated to be due to impaired immunological response and lower vaccine efficacy [22]; however, outcomes of acute infections related admissions in a hospital cohort have identified an increased survival with overweight or obesity suggesting the potential protective effects of low grade inflammation, immune activation, and increased energy reserves against infections [23].

In contrast to the increasing trend of overweight and obesity, the prevalence of underweight has reduced in our cohort compared to the background population (23.9% versus 15.4%) [10]. Aforementioned regional study conducted in Colombo reported even lower underweight prevalence (7.7%) [12]. Similar to obesity, underweight is also identified as a significant risk factor for in-hospital mortality [5, 24, 25], infections [26], and CVDs [27]. We failed to identify any mortality associations, but the prevalence of acute infections was significantly increased, whilst the prevalence of CVDs and CVD risk factors was decreased in patients with underweight compared to normal. Of note, both males and females with underweight had increased risk of CVDs compared to their normal-weight counterparts in subgroup analysis. There is a significant sparsity of evidence on CVDs and other metabolic risk factors among patients with underweight given the fact that major CVD risk factor prevalence studies were conducted almost exclusively in Caucasian populations where underweight prevalence is significantly low.

WHO recommends lower BMI cut-offs to trigger public health actions for Asians as they appear to have a different risk profile [8]. Although we observed a noticeable rise in the prevalence of overweight and obesity in ACs, no significant increment in the risk of mortality, morbidity, and in-hospital complications was noted. In fact, the length of hospital stay was decreased in the generalized obesity group. Additionally, the increased prevalence of CVD risk factors among patients with overweight was no longer statistically significant. Hence, periodic review of population cut-offs might be important, as the association between the BMI and comorbidities is liable to change over time with the dynamic environmental and nutritional transition.

Finally, in the light of our results, it is evident that overweight and obesity have a significant impact on hospital outcomes and existing evidence is inconsistent and lacks generalizability. Given the notable differences in body fat distribution, enhanced metabolic risk profiles with relatively low prevalence of overweight or obesity, and double burden of malnutrition and diseases among South Asians, the importance of large prospective studies to accurately gauge this problem is highlighted. In addition to aforementioned limitations, retrospective nature of the study, interindividual variations in clinical diagnoses, and anthropometric measurements are noted. Nonstandardized recording of patient data and paper-based record systems are significant challenges of conducting retrospective studies in resource-limited settings. However, we undertook robust steps to reduce data variability (Methods section). Additionally, being conducted in the largest referral center of the country adds to the generalizability, and including all the patients admitting with acute medical illnesses consecutively helped to minimize the selection bias. Including patients with a broad range of medical conditions and relatively large sample size compared to the reported single-center studies are other strengths of our study.

## 5. Conclusions

The prevalence of overweight, generalized obesity, and central obesity among hospital admissions is high with obesity being a risk factor for in-hospital all-cause mortality and AKI. There was a significant gender disparity in overweight or obesity prevalence and its associations. Acute infections were the leading cause of admissions followed by acute CVDs and extremes of body weight appear to have inverse associations with regard to prevalence of these conditions. The importance of larger prospective studies to further explore these associations in South Asian populations with distinct characteristics is highlighted.

## Figures and Tables

**Table 1:** Baseline characteristics.

Characteristics	Total (*n* = 2.128)	Male (*n* = 1.057)	Female (*n* = 1.071)	*p* value
Median age (years)	54 (IQR 42, 67)	54 (IQR 41, 67)	54 (IQR 43, 68)	0.41
Median BMI (kg/m^2^)	23 (IQR 20, 26)	22 (IQR 20, 26)	24 (IQR 20, 28)	<0.0001
Median WC (cm)	81 (IQR 68, 92)	81 (IQR 70, 97)	83 (IQR 69, 95)	0.011

*p* values: males versus females; BMI: body mass index; WC: waist circumference.

**Table 2:** In-hospital morbidity of the cohort (international body mass index/waist circumference cut-offs).

In-hospital morbidity	Total cohort (*n* = 2,128)	Classification as per international cut-offs [as per Asian cut-offs]												
		BMI group (*n* = 1,500)							WC group (*n* = 628)			Either GO/CO *n* = 1,541^!^ [*n* = 1,429]^!!^		
		NW *n* = 757 [*n* = 511]	UW *n* = 230 [*n* = 230]	OW *n* = 352 [*n* = 467]	GO *n* = 156 [*n* = 290]	*p* value^*∗*^	*p* value^#^	*p* value^	Low risk *n* = 449 [*n* = 329]	CO *n* = 179 [*n* = 299]	*p* value	Normal *n* = 1,206 [*n* = 840]	GO/CO *n* = 335 *n* = 589]	*p* value
AKI in hospital	35 (1.6%)	9 (1.2%) [7 (1.4%)]	3 (1.3%)	4 (1.1%) [5 (1.1%)]	2 (1.3%) [3 (1.0%)]	0.889 [0.933]	0.94 [0.671]	0.923 [0.681]	6 (1.3%) [5 (1.5%)]	11 (6.1%) [12 (4%)]	0.001 [0.054]	15 (1.2%) [12 (1.4%)]	13 (3.9%) [15 (2.5%)]	0.001 [0.126]
HAI	39 (1.8%)	10 (1.3%) [8 (1.6%)]	2 (0.9%)	1 (0.3%) [3 (0.6%)]	2 (1.3%) [2 (0.7%)]	0.584) [0.441]	0.105 [0.171]	0.969 [0.283]	18 (4%) [13 (4%)]	6 (3.4%) [11 (3.7%)]	0.698 [0.859]	28 (2.3%) [21 (2.5%)]	8 (2.4%) [13 (2.2%)]	0.943 [0.721]
Pressure ulcer	7 (0.3%)	1 (0.1%) [1 (0.2%)]	−	1 (0.3%) [1 (0.2%)]	−[−]	−[−]	0.579 [0.949]	−[−]	3 (0.7%) [3 (0.9%)]	2 (1.1%) [2 (0.7%)	0.578 [0.732]	4 (0.3%) [4 (0.5%)]	2 (0.6%) [2 (0.3%)]	0.49 [0.694]
ICU admissions	11 (0.5%)	1 (0.1%) [−]	4 (1.7%)	1 (0.3%) [1 (0.2%)]	1 (0.6%) [2 (0.7%)]	0.003 [−]	0.579 [−]	0.216 [−]	2 (0.4%) [2 (0.6%)]	2 (1.1%) [2 (0.7%)]	0.339 [0.924]	3 (0.2%) [2 (0.2%)]	3 (0.9%) [4 (0.7%)]	0.093 [0.204]
The median number of days in ICU	2 (IQR 2, 2)	1 (1, 1) [−]	3 (2, 11)	1 (1, 1) [1 (1, 1)]	4 (4, 4) [3 (1, 4)]	0.4 [−]	1.00 [−]	1.00 [−]	4 (2, 5) [4 (2, 5)]	6 (2, 9) [6 (2, 9)]	0.667 0.667	2 (1, 5) [4 (2, 5)]	4 (2, 9) [3 (2, 7)]	0.4 [1.0]
Median length of hospital stay	4 (2, 6)	3 (2, 6) [4 (2, 6)]	4 (2, 6)	3 (2, 5) [3 (2, 5)]	3 (2, 5) [3 (2, 5)]	0.413 [0.888]	0.103 [0.064]	0.252 [0.014]	4 (3, 7) [4 (2, 7)]	4 (2, 8) [4 (3, 7)]	0.96 0.718	4 (2, 6) [4 (3, 6)]	4 (2, 7) [4 (2, 6)]	0.942 [0.165]

AKI: acute kidney injury, BMI: body mass index, CO: central obesity, GO: generalized obesity, HAI: hospital acquired infections, ICU: intensive care units, NW: normal weight, OW: overweight, UW: underweight, WC: waist circumference; !: total of either GO/CO and normal BMI/WC in international cut-offs, !!: total of either GO/CO and normal BMI/WC in Asian cut-offs, ^*∗*^*p* values: UW group versus NW group, ^#^*p* values: OW group versus NW group, ^^^*p* values: GO group versus NW group.

**Table 3:** Prevalence of body mass index/waist circumference categories among acute diseases and comparison of acute disease prevalence between “normal” and “high risk” BMI/WC groups.

Disease category	Total (*n* = 2128)	Classification as per international cut-offs [as per Asian cut-offs]						*p* values				
		UW *n* = 232	NW *n* = 759 [*n* = 511]	OW *n* = 353 [*n* = 467]	GO *n* = 156 [*n* = 290]	L-WC *n* = 449 [*n* = 329]	CO *n* = 179 [*n* = 299]	UW^*∗*^	OW^*∗*^	GO^*∗*^	CO^#^	GO/CO ^
Cardiovascular disease	552 (25.9%)	32 (9.0%) [32 (9.0%)]	171 (48%) [109 (30.6%)]	106 (29.8%) [131 (36.8%)]	47 (13.2%) [84 (23.6%)]	136 (24.6%) [97 (17.6%)]	60 (10.9%) [99 (17.9%)]	0.004 [0.015]	0.007 [0.015]	0.040 [0.015]	0.430 [0.327]	0.018 [0.006]
Diabetes related	99 (4.7%)	8 (12.3%) [8 (12.3%)]	34 (52.3%) [22 (33.8%)]	17 (26.2%) [25 (38.5%)]	6 (9.2%) [10 (15.4%)]	26 (26.3%) [16 (16.2%)]	8 (8.1%) [18 (19.2%)]	0.505 [0.582]	0.802 [0.444]	0.720 [0.552]	0.569 [0.522]	0.547 [0.838]
GI/liver disease	76 (3.6%)	6 (12.8%) [6(12.8%)]	30 (63.8%) [20 (42.6%)]	7 (14.9%) [15 (31.9%)]	4 (8.5%) [6 (12.8%)]	26 (34.2%) [20 (26.3%)]	3 (3.9%) [9 (11.8%)]	0.337 [0.361]	0.088 [0.555]	0.401 [0.157]	0.027 [0.067]	0.037 [0.032]
Hematological diseases	28 (1.3%)	2 (9.5%) [2 (9.5%)]	11 (52.4%) [5 (23.8%)]	6 (28.6%) [8 (38.1%)]	2 (9.5%) [6 (28.6%)]	6 (21.4%) [3 (10.7%)]	1 (3.6%) [4 (14.3%)]	0.497 [0.879]	0.751 [0.316]	0.870 [0.202]	0.402 [0.612]	0.462 [0.214]
Infections	944 (44.4%)	136 (19.2%) [136 (19.3%)]	372 (52.4%) [265 (37.1%)]	138 (19.4%) [191 (26.8%)]	64 (9%) [120 (16.8%)]	166 (17.6%) [125 (13.2%)]	64 (6.8%) [105 (11.1%)]	0.008 [0.053]	0.002 [0.001]	0.065 [0.004]	0.775 [0.455]	0.036 [0.002]
Neurological/MSK diseases	138 (6.5%)	14 (15.5%) [14 (15.1%)]	40 (43.0%) [23 (24.7%)]	28 (30.1%) [33 (35.5%)]	11 (11.8%) [23 (24.7%)]	28 (20.3%) [20 (14.5%)]	17 (12.3%) [25 (18.1%)]	0.639 [0.373]	0.084 [0.085]	0.381 [0.045]	0.153 [0.268]	0.068 [0.021]
Respiratory disease	109 (5.1%)	19 (26.4%) [19 (26.0%)]	29 (40.3%) [19 (26.0%)]	12 (16.7%) [19 (26%)]	12 (16.7%) [16 (21.9%)]	30 (27.5%) [28 (25.7%)]	6 (5.5%) [8 (7.3%)]	0.006 [0.010]	0.729 [0.777]	0.034 [0.231]	0.105 [0.002]	0.721 [0.193]
Renal disease	71 (3.3%)	1 (2.4%) [1 (2.4%)]	24 (58.5%) [15 (36.6%)]	15 (36.6%) [20 (48.8%)]	1 (2.4%) [5 (12.2%)]	15 (21.1%) [11 (15.5%)]	15 (21.1%) [19 (26.8%)]	0.021 [0.029]	0.359 [0.257]	0.078 [0.291]	0.008 [0.077]	0.178 [0.321]
Miscellaneous diseases	164 (7.7%)	16 (13.9%) [16 (13.9%)]	59 (51.3%) [40 (34.8%)]	27 (23.5%) [34 (29.6%)]	13 (11.3%) [25 (21.7%)]	40 (24.4%) [27 (16.5%)]	9 (5.5%) [22 (13.4%)]	0.675 [0.656]	0.943 [0.747]	0.820 [0.693]	0.102 [0.692]	0.323 [0.998]

CO: central obesity, GO: generalized obesity, L-WC: low risk waist circumference, MSK: musculoskeletal, NW: normal weight, OW: overweight, UW: underweight. ^*∗*^*p* values derived in comparison to normal weight, ^#^*p* values derived in comparison to low-risk WC, and ^^^*p* values in comparison to normal BMI/WC group.

**Table 4:** Prevalence of body mass index/waist circumference categories among chronic diseases and comparison of chronic disease prevalence between “normal” and “high risk” body mass index/waist circumference groups.

Disease category	Total (*n* = 2128)	Classification as per international cut offs [as per Asian cut-offs]						*p* values				
		UW *n* = 232	NW *n* = 759 [*n* = 511]	OW *n* = 353 [*n* = 467]	GO *n* = 156 [*n* = 290]	L-WC *n* = 449 [*n* = 329]	CO *n* = 179 [*n* = 299]	UW^*∗*^	OW^*∗*^	GO^*∗*^	CO^#^	GO/CO ^
Dyslipidemia	342 (16.1%)	16 (6.3%) [16 (6.2%)]	129 (50.4%) [84 (32.7%)]	68 (26.6%) [88 (34.2)]	43 (16.8%) [69 (26.8%)]	51 (14.9%) [38 (11.1%)]	34 (9.9%) [47 (13.7%)]	<0.001 [<0.001]	0.356 [0.324]	0.002 [0.011]	0.010 [0.119]	<0.001 [0.009]
Heart failure	131 (6.2%)	11 (13.3%) [11 (13.3%)]	41 (49.4%) [29 (34.9%)]	21 (25.3%) [29 (34.9)]	10 (12%) [14 (16.9%)]	36 (27.5%) [28 (21.4%)]	12 (9.2%) [20 (15.3%)]	0.706 [0.601]	0.711 [0.724]	0.622 [0.609]	0.576 [0.391]	0.904 [0.440]
Hypertension	848 (39.9%)	50 (9.3%) [50 (9.3%)]	243 (45.3%) [163 (30.4%)]	151 (28.2%) [172 (32%)]	92 (17.2%) [152 (28.3%)]	194 (22.9%) [138 (16.3%)]	117 (13.8%) [173 (20.4%)]	0.003 [0.004]	<0.001 [0.104]	<0.001 [<0.001]	<0.001 [<0.001]	<0.001 [<0.001]
IHD	436 (20.5%)	31 (10.8%) [31(10.8%)]	137 (47.6%) [90 (31.3%)]	83 (28.8%) [102 (35.4%)]	37 (12.8%) [65 (22.6%)]	96 (22%) [66 (15.1%)]	52 (11.9%) [82 (18.8%)]	0.103 [0.146]	0.033 [0.096]	0.104 [0.015]	0.041 [0.030]	0.004 [0.004]
Osteoarthritis	41 (1.9%)	—	9 (34.6%) [5 (19.2%)]	10 (38.5%) [10 (38.5%)]	7 (26.9%) [11 (42.3%)]	8 (19.5%) [4 (9.8%)]	7 (17.1%) [11 (26.8%)]	0.097 [0.130]	0.050 [0.141]	0.004 [0.006]	0.115 [0.043]	0.001 [0.001]
T2DM	836 (39.3%)	37 (6.8%) [37 (6.8%)]	283 (51.7%) [191 (34.9%)]	150 (27.4%) [186 (33.9%)]	77 (14.1%) [134 (24.5%)]	177 (21.2%) [119 (14.2%)]	111 (13.3%) [169 (20.2%)]	<0.001 [<0.001]	0.097 [0.431]	0.005 [0.014]	<0.001 [<0.001]	<0.001 [<0.001]

CO: central obesity, GO: generalized obesity, IHD: ischemic heart disease, L-WC: low risk waist circumference, NW: normal weight, OW: overweight, T2DM: type-2 diabetes mellitus, UW: underweight. ^*∗*^*p* values derived in comparison to normal weight, ^#^*p* values derived in comparison to low-risk WC, and ^*p* values in comparison to normal BMI/WC group.

## Data Availability

All data generated or analyzed during this study are included in this article and its Supplementary Materials files. Further enquiries can be directed to the corresponding author.

## Supplementary Materials

Supplementary Materials Supplementary File 1: tables. Supplementary File 2: questionnaire. Supplementary File 3: STROBE statement—checklist of items that should be included in reports of cohort studies.
