# Supplementary material for: Influence of Overweight and Obesity on Morbidity and Mortality among Hospitalized Patients in Sri Lanka: A Single-Center Analysis
Source: J Obes. 2022 Aug 18;2022:9172365. doi: 10.1155/2022/9172365 (PMC9411002; doi:10.1155/2022/9172365)
Supplement: Supplementary Materials — Supplementary File 1: tables. Supplementary File 2: questionnaire. Supplementary File 3: STROBE statement—checklist of items that should be included in reports of cohort studies. [file 9172365.f1.zip › 9172365.f1/Supplementary file 1 (2).docx]

**Supplementary file 1**

1. Table 1: International, Asian, Sri Lankan body mass index classifications

| Category | International  Classification^*^( Kg/m^2^) | Asian population BMI cut offs^*^ (Kg/m^2^) | Sri Lankan BMI cut offs^±^ (Kg/m^2^) |
| --- | --- | --- | --- |
| Underweight | <18.5 | <18.5 | <18.5 |
| Normal | 18.5-24.9 | 18.5-23 | 18.5-22.9 |
| Over weight | 25.0-29.9 | Increased risk: 23-27.5 | 23-24.9 |
| Obesity 1  Obesity 11 | 30.0-34.5  35.0-39.9 | Higher high risk: ≥27.5 | 25-29.9  30-334.9 |
| Extreme obesity | >40 |  | ≥35 |

Adopted from WHO expert consultation (8), ± adopted from clinical guidelines, management of obesity, The Endocrine society of Sri Lanka(33)

1. Table 2: Waist circumference classification

| Category | WC in international cut offs | | WC in Asian cut offs | |
| --- | --- | --- | --- | --- |
|  | **Males** | **Females** | **Males** | **Females** |
| High risk | ≥102 cm | ≥88 cm | ≥90 cm | ≥80 cm |
| Low risk | <102 cm | <88 cm | <90 cm | <80cm |

Adopted from WHO expert consultation (8)

1. Table 3: Acute medical conditions and composite variables

| Disease Category | Total | Male | Female |
| --- | --- | --- | --- |
| Acute Disease (Composite variable) | | | |
| Cardiovascular disease   1. ACS 2. Acute heart failure 3. Acute stroke 4. Hypertensive emergencies | 1. 309(14.49%) 2. 74(3.47%) 3. 83(3.89%) 4. 38(1.78%) | 172 (55.66%)  37 (50.0%)  46 (55.42%)  11 (28.95%) | 137 (44.34%)  37 (50.0%)  37 (44.58%)  27 (71.05%) |
| Respiratory disease   1. Acute URTI 2. Acute LRTI 3. BA exacerbation 4. COPD exacerbation | 1. 10(0.47%) 2. 153(7.18%) 3. 46(2.16%) 4. 58(2.72%) | 5 (50.0%)  68 (44.4%)  9 (19.57%)  51 (87.93%) | 5 (50.0%)  85 (55.56%)  37 (80.43%)  7 (12.07%) |
| Acute presentations related to DM   1. Hyperglycemic emergencies 2. Hypoglycemic emergencies | 1. 85(3.99%) 2. 17(0.80%) | 34 (40.0%)  6 (35.29%) | 51 (60.0%)  11 (64.71%) |
| Gastrointestinal and liver diseases   1. Acute UGI bleeding 2. GORD 3. Acute intestinal infections 4. Alcoholic liver disease | 1. 16 (0.75%) 2. 26 (1.22%) 3. 23 (1.08%) 4. 34 (1.59%) | 12 (75.0%)  10 (38.46%)  12 (52.17%)  27 (79.41%) | 4 (25.0%)  16 (61.54%)  11 (47.83%)  7 (20.59%) |
| Renal diseases   1. AKI on CKD 2. UTI 3. Urolithiasis 4. Pyelonephritis 5. Acute urine retention | 1. 73 (3.42%) 2. 110 (5.16%) 3. 4 (0.19%) 4. 50 (2.35%) 5. 2 (0.09%) | 34 (46.58%)  46 (41.82%)  2 (50.0%)  17 (34.0%)  0 (0.0%) | 39 (53.42%)  64 (58.18%)  2 (50.0%)  33 (66.0%)  2 (100.0%) |
| Infections   1. TB 2. Infection (bacterial) (Lepto/I Endocarditis) 3. Dengue Fever/ Haemorrhagic 4. Viral infections non specific 5. Meningoencephalitis/ Encephalitis 6. Infection (bacterial) Cellulitis | 1. 16 (0.75%) 2. 44 (2.06%) 3. 274 (12.85%) 4. 180 (8.44%) 5. 20 (0.94%) 6. 50 (2.35%) | 12 (75.0%)  35 (79.55%)  138 (50.36%)  102 (56.67%)  8 (40.00%)  24 (48.00%) | 4 (25.0%)  9 (20.45%)  136 (49.64%)  78 (43.33%)  12 (60.00%)  26 (52.00%) |
| Hematological diseases   1. Deep venous thrombosis 2. Anaemia 3. Purpura and other hemorrhagic conditions | 1. 5(0.23%) 2. 19 (0.89%) 3. 5 (0.23%) | 2 (40.00%)  10 (52.63%)  2 (40.00%) | 3 (60.00%)  9 (47.37%)  3 (60.00%) |
| Neurological/ musculoskeletal diseases   1. Neuropathy 2. Seizure 3. Headache 4. GBS 5. Disorders of vestibular functions 6. Arthritis 7. Back pain/ Neck pain 8. Syncope | 1. 14 (0.66%) 2. 15 (0.70%) 3. 26 (1.22%) 4. 6 (0.28%) 5. 20 (0.94%) 6. 20 (0.94%) 7. 39 (1.83%) 8. 5 (0.23%) | 10 (71.43%)  14 (93.33%)  9 (34.62%)  3 (50.00%)  5 (25.00%)  8 (40.0%)  25 (64.10%)  3 (60.00%) | 4 (28.57%)  1 (6.67%)  17 (65.38%)  3 (50.00%)  15 (75.00%)  12 (60.00%)  14 (35.90%)  2 (40.00%) |
| Miscellaneous   1. PUO 2. Malignancy 3. Valvular heart disease,Acute/chronic rheumatic heart disease 4. Dermatitis and eczema 5. Non specific chest pain 6. Abnormal findings of blood chemistry 7. Poisoning | 1. 12 (0.56%) 2. 15 (0.70%) 3. 4 (0.19%) 4. 16 (0.75%) 5. 71 (3.33%) 6. 12 (0.56%) 7. 31 (1.45%) | 7 (58.33%)  6 (40.00%)  2 (50.00%)  8 (50.00%)  18 (25.35%)  5 (41.67%)  15 (48.39%) | 5 (41.67%)  9 (60.00%)  2 (50.00%)  8 (50.00%)  53 (74.65%)  7 (58.33%)   1. 51.61%) |

1. Table 4: Acute medical conditions reported in the hospital cohort

| Disease category | Total (n=2128) | Males  (n=1,057) | Females  (n=1,071) | P values |
| --- | --- | --- | --- | --- |
| Cardiovascular disease | 552 (25.9%) | 294 (53.3%) | 258 (46.7%) | **0.05** |
| Diabetes related | 99 (4.7%) | 39 (39.4%) | 60 (60.6%) | **0.036** |
| Gastrointestinal and liver disease | 76 (3.6%) | 49 (64.5%) | 27 (35.5%) | **0.009** |
| Hematological diseases | 28 (1.3%) | 14 (50%) | 14 (50%) | 0.972 |
| Infections | 944 (44.4%) | 473 (50.1%) | 471 (49.9%) | 0.72 |
| Neurological/ MSK diseases | 138 (6.5%) | 62 (44.9%) | 76 (55.1%) | 0.249 |
| Respiratory disease | 109 (5.1%) | 65 (59.6%) | 44 (40.4%) | **0.03** |
| Renal disease | 71 (3.3%) | 32 (45.1%) | 39 (54.9%) | 0.43 |
| Miscellaneous diseases | 164 (7.7%) | 66 (40.2%) | 98 (59.8%) | **0.012** |

P value males vs. females; MSK - Musculoskeletal

1. Acute diseases prevalence in high body mass index groups and waist circumference groups in comparison to ‘normal’ in international cut-offs
   1. Normal weight (BMI 18.5-24.9) Vs over weight (BMI 25-29.9)

| Disease category | NW(BMI 18.5-24.9)  N=757 | | | OW(BMI 25-29.9)  N=352 | | | P value | | |
| --- | --- | --- | --- | --- | --- | --- | --- | --- | --- |
|  | T | M | F | T | M | F | *T** | *M^#^* | *F^* |
| Cardiovascular disease | 171(22.6%) | 106(62%) | 65(38%) | 106(30.1%) | 61(57.5%) | 45(42.5%) | *0.007* | *0.009* | *0.158* |
| Diabetes related | 34(4.5%) | 13(38.2%) | 21(61.8%) | 17(4.8%) | 4(23.5%) | 13(76.5%) | *0.802* | *0.630* | *0.703* |
| Gastrointestinal and liver disease | 30(4%) | 21(70%) | 9(30%) | 7(2%) | 2(28.6%) | 5(71.4%) | *0.088* | *0.03* | *0.968* |
| Hematological diseases | 11(1.5%) | 7(63.6%) | 4(36.4%) | 6(1.7%) | 1(16.7%) | 5(83.3%) | *0.751* | *0.307* | *0.197* |
| Infections | 372(49.1%) | 212(57%) | 160(43%) | 138(39.2%) | 68(49.3%) | 70(50.7%) | *0.002* | *0.024* | *0.41* |
| Neurological/ MSK diseases | 40(5.3%) | 21(52.5%) | 19(47.5%) | 28(8%) | 12(42.9%) | 16(57.1%) | *0.084* | *0.319* | *0.178* |
| Respiratory disease | 29(3.8%) | 15(51.7%) | 14(48.3%) | 12(3.4%) | 8(66.7%) | 4(33.3%) | *0.729* | *0.514* | *0.24* |
| Renal disease | 24(3.2%) | 10(41.7%) | 14(58.3%) | 15(4.3%) | 9(60%) | 6(40%) | *0.359* | *0.068* | *0.619* |
| Miscellaneous diseases | 59(7.8%) | 27(45.8%) | 32(54.2%) | 27(7.7%) | 9(33.3%) | 18(66.7%) | *0.943* | *0.609* | *0.903* |

T:Total, M:Male, F:Female, NW: normal weight, OW: over weight, *T: p value derived from total of OW group compared with NW group, ^#^M: p value derived from males in the OW group compared with NW group, ^F: p value derived from females in the OW group compared with NW group

- 1. Normal weight (BMI 18.5-24.9) Vs Generalized obesity(GO)

| Disease categories | NW(BMI 18.5-24.9)  N=757 | | | GO (BMI≥30)  N=156 | | | P values | | |
| --- | --- | --- | --- | --- | --- | --- | --- | --- | --- |
|  | T | M | F | T | M | F | T* | M^#^ | F^ |
| Cardiovascular disease | 171(22.6%) | 106(62%) | 65(38%) | 47(30.1%) | 19(40.4%) | 28((59.6%) | *0.04* | *0.074* | *0.107* |
| Diabetes related | 34(4.5%) | 13(38.2%) | 21(61.8%) | 6(3.8%) | 2(33.3%) | 4(66.7%) | *0.72* | *0.761* | *0.346* |
| Gastrointestinal and liver disease | 30(4%) | 21(70%) | 9(30%) | 4(2.6%) | 1(25%) | 3(75%) | *0.401* | *0.326* | *0.921* |
| Hematological diseases | 11(1.5%) | 7(63.6%) | 4(36.4%) | 2(1.3%) | 1(50%) | 1(50%) | *0.87* | *0.885* | *0.841* |
| Infections | 372(49.1%) | 212(57%) | 160(43%) | 64(41%) | 22(34.4%) | 42(65.6%) | *0.065* | *0.295* | *0.171* |
| Neurological/ MSK diseases | 40(5.3%) | 21(52.5%) | 19(47.5%) | 11(7.1%) | 2(18.2%) | 9(81.8%) | *0.381* | *0.725* | *0.284* |
| Respiratory disease | 29(3.8%) | 15(51.7%) | 14(48.3%) | 12(7.7%) | 2(16.7%) | 10(83.3%) | *0.034* | *0.91* | *0.034* |
| Renal disease | 24(3.2%) | 10(41.7%) | 14(58.3%) | 1(0.6%) | 1(100%) | - | *0.078* | *0.844* | *-* |
| Miscellaneous diseases | 59(7.8%) | 27(45.8%) | 32(54.2%) | 13(8.3%) | 4(30.8%) | 9(69.2%) | *0.820* | *0.715* | *0.77* |

T:Total, M:Male, F:Female, NW: normal weight, GO: generalized obesity, *T: p value derived from total of GO group compared with NW group, ^#^M: p value derived from males in the GO group compared with NW group, ^F: p value derived from females in the GO group compared with NW group

- 1. Low risk WC (<102 in M, <88 in F) Vs Central obesity (WC≥102 in M, ≥88 in F)

| Disease category | Low risk [WC<102(M), <88(F)]  N=449 | | | CO[WC≥102(M), ≥88(F)]  N=179 | | | P values | | |
| --- | --- | --- | --- | --- | --- | --- | --- | --- | --- |
|  | T | M | F | T | M | F | T* | M^#^ | F^ |
| Cardiovascular disease | 136(30.3%) | 81(59.6%) | 55(40.4%) | 60(33.5%) | 7(11.7%) | 53(88.3%) | 0.430 | 0.84 | 0.311 |
| Diabetes related | 26(5.8%) | 16(61.5%) | 10(38.5%) | 8(4.5%) | - | 8(100%) | *0.569* | *0.211* | *0.957* |
| Gastrointestinal and liver disease | 26(5.8%) | 19(73.1%) | 7(26.9%) | 3(1.7%) | 1(33.3%) | 2(66.7%) | *0.027* | *0.565* | *0.163* |
| Hematological diseases | 6(1.3%) | 3(50%) | 3(50%) | 1(0.6%) | - | 1(100%) | *0.402* | *0.597* | *0.417* |
| Infections | 166(37%) | 92(55.4%) | 74(44.6%) | 64(35.8%) | 10(15.6%) | 54(84.4%) | *0.775* | *0.539* | *0.41* |
| Neurological/ MSK diseases | 28(6.2%) | 18(64.3%) | 10(35.5%) | 17(9.5%) | 2(11.8%) | 15(88.2%) | *0.153* | *0.796* | *0.119* |
| Respiratory disease | 30(6.7%) | 22(73.3%) | 8(26.7%) | 6(3.4%) | 2(33.3%) | 4(66.7%) | 0.105 | 0.983 | 0.406 |
| Renal disease | 15(3.3%) | 8(53.3%) | 7(46.7%)2 | 15(8.4%) | 4(26.7%) | 11(73.3%) | 0.008 | 0.002 | 0.16 |
| Miscellaneous diseases | 40(8.9%) | 17(42.5%) | 3(57.5%) | 9(5.0%) | 1(11.1%) | 8(88.9%) | 0.102 | 0.648 | 0.024 |

T:Total, M:Male, F:Female, CO: Central obesity, *T: p value derived from total of CO group compared with low risk group, ^#^M: p value derived from males in the CO group compared with low risk group, ^F: p value derived from females in the CO group compared with low risk group

- 1. Normal BMI/WC Vs either generalize/central obesity

| Disease category | Normal BMI (18.5-24.9)/WC [WC<102(M), <88(F)]  N=1206 | | | \|  \| GO (BMI≥30)/ CO[WC≥102(M), ≥88(F)]  N=355 \| \| --- \| --- \| | | | P values | | |
| --- | --- | --- | --- | --- | --- | --- | --- | --- | --- | --- | --- |
|  | T | M | F | T | M | F | T* | M^#^ | F^ |
| Cardiovascular disease | 307(25.5%) | 187(60.9%) | 120(39.1%) | 107(31.9%) | 26(24.3%) | 81(75.7%) | 0.018 | 0.206 | 0.013 |
| Diabetes related | 60(5%) | 29(48.3%) | 31(51.7%) | 14(4.2%) | 2(14.3%) | 12(85.7%) | *0.547* | *0.581* | *0.451* |
| Gastrointestinal and liver disease | 56(4.6%) | 40(71.4%) | 16(28.6%) | 7(2.1%) | 2(28.6%) | 5(71.4%) | *0.037* | *0.244* | *0.356* |
| Hematological diseases | 17(1.4%) | 10(58.8%) | 7(41.2%) | 3(0.9%) | 1(33.3%) | 2(66.7%) | *0.462* | *0.919* | *0.483* |
| Infections | 538(44.6%) | 304(56.5%) | 234(43.5%) | 128(38.2%) | 32(25%) | 96(75%) | *0.036* | *0.697* | *0.039* |
| Neurological/ MSK diseases | 68(5.6%) | 39(57.4%) | 29(42.6%) | 28(8.4%) | 4(14.3%) | 24(85.7%) | *0.068* | *0.875* | *0.052* |
| Respiratory disease | 59(4.9%) | 37(62.2%) | 22(37.3%) | 18(5.4%) | 4(22.2%) | 14(77.8%) | 0.721 | 0.957 | 0.456 |
| Renal disease | 39(3.2%) | 18(46.2%) | 21(53.8%) | 16(4.8%) | 5(31.3%) | 11(68.8%) | 0.178 | 0.057 | 0.882 |
| Miscellaneous diseases | 99(8.2%) | 44(44.4%) | 55(55.6%) | 22(6.6%) | 5(22.7%) | 17(77.3%) | 0.323 | 0.961 | 0.071 |

T:Total, M:Male, F:Female, GO: Generalized obesity, CO: central obesity, *T: p value derived from total of Generalized/central obesity group compared with Normal BMI/WC group, ^#^M: p value derived from males in the Generalized/central obesity group compared with Normal BMI/WC group, ^F: p value derived from females in the Generalized/central obesity group compared with Normal BMI/WC group

1. Table 6: Acute diseases prevalence in high body mass index groups and waist circumference groups in comparison to ‘normal’ in Asian cut-offs.
   1. Normal weight (BMI 18.5-23) Vs overweight/ increased risk (BMI 23-27.5)

| Disease category | NW(BMI 18.5-23)  N=232 | | | OW(BMI 23-27.5)  N=467 | | | P value | | |
| --- | --- | --- | --- | --- | --- | --- | --- | --- | --- |
|  | T | M | F | T | M | F | *T** | *M^#^* | *F^* |
| Cardiovascular disease | 109(21.3%) | 71(65.1%) | 38(34.9%) | 131(28.1%) | 75(57.3%) | 56(42.7%) | *0.015* | *0.137* | *0.032* |
| Diabetes related | 22(4.3%) | 8(36.4%) | 14(63.6%) | 25(5.4%) | 9(36%) | 16(64%) | *0.444* | *0.567* | *0.686* |
| Gastrointestinal and liver disease | 20(3.9%) | 16(80%) | 4(20%) | 15(3.2%) | 7(46.7%) | 8(53.3%) | *0.555* | *0.123* | *0.235* |
| Hematological diseases | 5(1%) | 2(40%) | 3(60%) | 8(1.7%) | 5(62.5%) | 3(37.5%) | *0.316* | *0.176* | *0.991* |
| Infections | 265(51.9%) | 152(57.4%) | 113(42.6%) | 191(40.9%) | 105(55%) | 86(45%) | *0.001* | *0.019* | *0.012* |
| Neurological/ MSK diseases | 23(4.5%) | 11(47.8%) | 12(52.2%) | 33(7.1%) | 18(54.4%) | 15(45.5%) | *0.085* | *0.077* | *0.535* |
| Respiratory disease | 19(3.7%) | 12(63.2%) | 7(36.8%) | 19(4.1%) | 9(47.4%) | 10(52.6%) | *0.777* | *0.759* | *0.446* |
| Renal disease | 15(2.9%) | 6(40%) | 9(60%) | 20(4.3%) | 11(55%) | 9(45%) | *0.257* | *0.118* | *0.984* |
| Miscellaneous diseases | 40(7.8%) | 18(45%) | 22(55%) | 34(7.3%) | 17(50%) | 17(50%) | *0.747* | *0.764* | *0.418* |

T:Total, M:Male, F:Female, NW: normal weight, OW: over weight, *T: p value derived from total of OW group compared with NW group, ^#^M: p value derived from males in the OW group compared with NW group, ^F: p value derived from females in the OW group compared with NW group

- 1. Normal weight (BMI 18.5-24.9) Vs generalized obesity/ Higher high risk BMI

| Disease categories | NW(BMI 18.5-23)  N=511 | | | GO (BMI≥27.5)  N=290 | | | P values | | |
| --- | --- | --- | --- | --- | --- | --- | --- | --- | --- |
|  | T | M | F | T | M | F | T* | M^#^ | F^ |
| Cardiovascular disease | 109(21.3%) | 71(65.1%) | 38(34.9%) | 84(29%) | 40(47.6%) | 44(52.4%) | 0.015 | 0.008 | 0.103 |
| Diabetes related | 22(4.3%) | 8(36.4%) | 14(63.6%) | 10(3.4%) | 2(20%) | 8(80%) | *0.552* | *0.631* | *0.368* |
| Gastrointestinal and liver disease | 20(3.9%) | 16(80%) | 4(20%) | 6(2.1%) | 1(16.7%) | 5(83.3%) | *0.157* | *0.048* | *0.547* |
| Hematological diseases | 5(1%) | 2(40%) | 3(60%) | 6(2.1%) | 2(33.3%) | 4(66.7%) | *0.202* | *0.288* | *0.538* |
| Infections | 265(51.9%) | 152(57.4%) | 113(42.6%) | 120(41.4%) | 46(38.3%) | 74(61.7%) | *0.004* | *0.127* | *0.022* |
| Neurological/ MSK diseases | 23(4.5%) | 11(47.8%) | 12(52.2%) | 23(7.9%) | 6(26.1%) | 17(73.9%) | *0.045* | *0.409* | *0.146* |
| Respiratory disease | 19(3.7%) | 12(63.2%) | 7(36.8%) | 16(5.5%) | 4(25%) | 12(75%) | 0.231 | 0.88 | 0.117 |
| Renal disease | 15(2.9%) | 6(40%) | 9(60%) | 5(1.7%) | 3(60%) | 2(40%) | 0.291 | 0.646 | 0.064 |
| Miscellaneous diseases | 40(7.8%) | 18(45%) | 22(55%) | 25(8.6%) | 5(20%) | 20(80%) | 0.693 | 0.584 | 0.787 |

T:Total, M:Male, F:Female, NW: normal weight, GO: generalized obesity, *T: p value derived from total of GO group compared with NW group, ^#^M: p value derived from males in the GO group compared with NW group, ^F: p value derived from females in the GO group compared with NW group

- 1. Low risk WC (<90 in M, <80 in F) Vs Central obesity (WC≥90 in M, ≥80 in F)

| Disease category | Low risk [WC<90(M), <80(F)] | | | CO[WC≥90(M), ≥80(F)] | | | P values | | |
| --- | --- | --- | --- | --- | --- | --- | --- | --- | --- |
|  | T | M | F | T | M | F | T* | M^#^ | F^ |
| Cardiovascular disease | 97(29.5%) | 57(58.8%) | 40(41.2%) | 99(33.1%) | 31(31.3%) | 68(68.7%) | *0.327* | *0.162* | *0.904* |
| Diabetes related | 16(4.9%) | 10(62.5%) | 6(37.5%) | 18(6%) | 6(33.6%) | 12(66.7%) | *0.522* | *0.475* | *0.708* |
| Gastrointestinal and liver disease | 20(6.1%) | 13(65%) | 7(35%) | 9(3%) | 7(77.8%) | 2(22.2% | *0.067* | *0.582* | *0.011* |
| Hematological diseases | 3(0.9%) | 2(66.7%) | 1(33.3%) | 4(1.3%) | 1(25%) | 3(75%) | *0.612* | *0.886* | *0.603* |
| Infections | 125(38%) | 77(61.6%) | 48(38.4%) | 105(35.1%) | 25(23.8%) | 80(76.2%) | *0.455* | *0.161* | *1* |
| Neurological/ MSK diseases | 20(6.1%) | 15(75%) | 5(25%) | 25(8.4%) | 5(20%) | 20(80%) | *0.268* | *0.642* | *0.061* |
| Respiratory disease | 28(8.5%) | 21(75%) | 7(25%) | 8(2.7%) | 3(37.5%) | 5(62.5%) | *0.002* | *0.055* | *0.129* |
| Renal disease | 11(3.3%) | 5(45.5%) | 6(54.5%) | 19(6.4%) | 7(36.8%) | 12(63.2%) | *0.077* | *0.026* | *0.708* |
| Miscellaneous diseases | 27(8.2%) | 13(48.1%) | 14(51.9%) | 22(7.4%) | 5(22.7%) | 17(77.3%) | *0.692* | *0.863* | *0.356* |

T:Total, M:Male, F:Female, CO: Central obesity, *T: p value derived from total of CO group compared with low risk group, ^#^M: p value derived from males in the CO group compared with low risk group, ^F: p value derived from females in the CO group compared with low risk group

- 1. Categories of acute diseases in the normal BMI/WC Vs Generalized/central obesity groups

| Disease category | Normal BMI (18.5-23)/WC [WC<90(M), <80(F)]  N=840 | | | \|  \| GO (BMI≥27.5)/ CO[WC≥90(M), ≥80(F)]  N=589 \| \| --- \| --- \| | | | P values | | |
| --- | --- | --- | --- | --- | --- | --- | --- | --- | --- | --- | --- |
|  | T | M | F | T | M | F | T* | M^#^ | F^ |
| Cardiovascular disease | 206(24.5%) | 128(62.1%) | 78(37.9%) | 183(31.1%) | 71(38.8%) | 112(61.2%) | *0.006* | *0.003* | *0.077* |
| Diabetes related | 38(4.5%) | 18(47.4%) | 20(52.6%) | 28(4.8%) | 8(28.6%) | 20(71.4%) | *0.838* | *0.736* | *0.656* |
| Gastrointestinal and liver disease | 40(4.8%) | 29(72.5%) | 11(27.5%) | 15(2.5%) | 8(53.3%) | 7(46.7%) | *0.032* | *0.384* | *0.211* |
| Hematological diseases | 8(1%) | 4(50%) | 4(50%) | 10(1.7%) | 3(30%) | 7(70%) | *0.214* | *0.374* | *0.494* |
| Infections | 390(46.4%) | 229(58.7%) | 161(41.3%) | 225(38.2%) | 71(31.6%) | 154(68.4%) | *0.002* | *0.03* | *0.034* |
| Neurological/ MSK diseases | 43(5.1%) | 26(60.5%) | 17(39.5%) | 48(8.1%) | 11(22.9%) | 37(77.1%) | *0.021* | *0.794* | *0.021* |
| Respiratory disease | 47(5.6%) | 33(70.2%) | 14(29.8%) | 24(4.1%) | 7(29.2%) | 17(70.8%) | *0.193* | *0.132* | *0.871* |
| Renal disease | 26(3.1%) | 11(42.3%) | 15(57.7%) | 24(4.1%) | 10(41.7%) | 14(58.3%) | *0.321* | *0.04* | *0.572* |
| Miscellaneous diseases | 67(8%) | 31(46.3%) | 36(53.7%) | 47(8%) | 10(21.3%) | 37(78.7%) | *0.998* | *0.609* | *0.623* |

T:Total, M:Male, F:Female, GO: Generalized obesity, CO: central obesity

*T: p value derived from total of Generalized/central obesity group compared with Normal BMI/WC group, ^#^M: p value derived from males in the Generalized/central obesity group compared with Normal BMI/WC group, ^F: p value derived from females in the Generalized/central obesity group compared with Normal BMI/WC group

1. Table 7: Acute diseases prevalence in underweight group in comparison to ‘normal’
   1. Normal weight (BMI 18.5-24.9) Vs Under-weight (BMI <18.5); international cut-offs

| Disease category | NW(BMI 18.5-24.9)  N=757 | | | *UW(BMI<18.5)*  *N=230* | | | *P values* | | |
| --- | --- | --- | --- | --- | --- | --- | --- | --- | --- |
|  | T | M | F | *T* | *M* | *F* | *T** | *M^#^* | *F^* |
| Cardiovascular disease | 171(22.6%) | 106(61.6%) | 65(38%) | 32(13.9%) | 20(62.5%) | 12(37.5%) | *0.004* | *0.039* | *0.053* |
| Diabetes related | 34(4.5%) | 13(38.2%) | 21(61.8%) | 8(3.5%) | 4(50%) | 4(50%) | *0.505* | *0.928* | *0.327* |
| Gastrointestinal and liver disease | 30(4%) | 21(70%) | 9(30%) | 6(2.6%) | 5(63.3%) | 1(16.7%) | *0.337* | *0.671* | *0.290* |
| Hematological diseases | 11(1.5%) | 7(63.6%) | 4(36.4%) | 2(0.9%) | 2(100%) | - | *0.497* | *0.977* | *-* |
| Infections | 372(49.1%) | 212(57%) | 160(43%) | 136(59.1%) | 67(49.3%) | 69(50.7%) | *0.008* | *0.429* | *0.002* |
| Neurological/ MSK diseases | 40(5.3%) | 21(52.5%) | 19(47.5%) | 14(6.1%) | 7(50%) | 7(50%) | *0.639* | *0.758* | *0.733* |
| Respiratory disease | 29(3.8%) | 15(51.7%) | 14(48.3%) | 19(8.3%) | 16(84.2%) | 3(15.8%) | *0.006* | *<0.0001* | *0.523* |
| Renal disease | 24(3.2%) | 10(41.7%) | 14(58.3%) | 1(0.4%) | - | 1(100%) | *0.021* | *-* | *0.107* |
| Miscellaneous diseases | 59(7.8%) | 27(45.9%) | 32(54.2%) | 16(7%) | 8(50%) | 8(50%) | *0.675* | *0.973* | *0.520* |

T:Total, M:Male, F:Female, NW: normal weight, UW: under weight, *T: p value derived from total of UW group compared with NW group, ^#^M: p value derived from males in the UW group compared with NW group, ^F: p value derived from females in the UW group compared with NW group, Normal weight (BMI 18.5-23) Vs Under-weight (BMI <18.5)

- 1. Normal weight (BMI 18.5-23) Vs Under-weight (BMI <18.5); Asian cut-offs

| Disease category | NW(BMI 18.5-23)  N=511 | | | *UW(BMI<18.5)*  *N=232* | | | *P values* | | |
| --- | --- | --- | --- | --- | --- | --- | --- | --- | --- |
|  | T | M | F | *T* | *M* | *F* | *T** | *M^#^* | *F^* |
| Cardiovascular disease | 109(21.3%) | 71(65.1%) | 38(34.9%) | 32(13.8%) | 20(62.5%) | 12(37.5%) | *0.015* | *0.06* | *0.149* |
| Diabetes related | 22(4.3%) | 8(36.4%) | 14(63.6%) | 8(3.4%) | 4(50%) | 4(50%) | *0.582* | *0.797* | *0.324* |
| Gastrointestinal and liver disease | 20(3.9%) | 16(80%) | 4(20%) | 6(2.6%) | 5(83.3%) | 1(16.7%) | *0.361* | *0.527* | *0.538* |
| Hematological diseases | 5(1%) | 2(40%) | 3(60%) | 2(0.9%) | 2(100%) | - | *0.879* | *0.380* | *0.224* |
| Infections | 265(51.9%) | 152(57.4%) | 113(42.6%) | 138(59.5%) | 68(49.3%) | 70(50.7% | *0.053* | *0.660* | *0.018* |
| Neurological/ MSK diseases | 23(4.5%) | 11(47.8%) | 12(52.2%) | 14(6%) | 7(50%) | 7(50%) | *0.373* | *0.399* | *0.701* |
| Respiratory disease | 19(3.7%) | 12(63.2%) | 7(36.8%) | 19(8.2%) | 16(84.2%) | 3(15.8%) | *0.01* | *0.001* | *0.847* |
| Renal disease | 15(2.9%) | 6(40%) | 9(60%) | 1(0.4%) | - | 1(100%) | *0.029* | *0.106* | *0.119* |
| Miscellaneous diseases | 40(7.8%) | 18(45%) | 22(55%) | 16(6.9%) | 8(50%) | 8(50%) | *0.656* | *0.927* | *0.451* |

T:Total, M:Male, F:Female, NW: normal weight, UW: under weight, *T: p value derived from total of UW group compared with NW group, ^#^M: p value derived from males in the UW group compared with NW group, ^F: p value derived from females in the UW group compared with NW group

1. Table 8: Categories of chronic diseases which are associated with obesity observed in the cohort

| Disease category | Total | Males | Females | P values* |
| --- | --- | --- | --- | --- |
| Dyslipidemia | 342(16.1%) | 115(33.6%) | 227(66.4%) | <0.0001 |
| Heart failure | 131(6.2%) | 81(61.8%) | 50(38.2%) | 0.004 |
| Hypertension | 848(39.9%) | 372(43.9%) | 476(56.1%) | <0.0001 |
| Ischemic heart disease | 436(20.5%) | 227(52.1%) | 209(47.9%) | 0.262 |
| Osteoarthritis | 41(1.9%) | 5(12.2%) | 36(87.8%) | <0.0001 |
| Type 2 diabetes mellitus | 836(39.3%) | 375(44.9%) | 461(55.1%) | <0.0001 |
| Venous thrombosis | 12(0.6%) | 1(8.3%) | 11(91.6%) | 0.004 |

1. Table 9: Chronic diseases prevalence in ‘ high risk’ body mass index groups and waist circumference groups in comparison to ‘normal’ in International cut-offs.
   1. Normal weight (BMI 18.5-24.9) Vs Over weight (BMI 25-29.9)

| Disease category | NW(BMI 18.5-24.9) | | | OW(BMI 25-29.9) | | | P values | | |
| --- | --- | --- | --- | --- | --- | --- | --- | --- | --- |
|  | T | M | F | T | M | F | *T | ^#^M | ^F |
| Dyslipidemia | 129(17%) | 55(42.6%) | 74(57.4%) | 68(19.3%) | 23(33.8%) | 45(66%.2) | *0.356* | *0.876* | *0.498* |
| Heart failure | 41(5.4%) | 31(75.6%) | 10(24.4%) | 21(6%) | 17(81%) | 4(19%) | *0.711* | *0.286* | *0.598* |
| Hypertension | 243(32.1%) | 121(49.8%) | 122(50.2%) | 151(42.9%) | 73(48.3%) | 78(51.7%) | *<0.0001* | *0.001* | *0.152* |
| Ischemic heart disease | 137(18.1%) | 82(59.9%) | 55(40.1%) | 83(23.6%) | 53(63.9%) | 30(36.1%) | *0.033* | *0.002* | *0.986* |
| Osteoarthritis | 9(1.2%) | 3(33.3%) | 6(66.7%) | 10(2.8%) | - | 10(100%) | *0.05* | *0.269* | *0.021* |
| Type 2 diabetes mellitus | 283(37.4%) | 142(50.2%) | 141(49.8%) | 150(42.6%) | 74(49.3%) | 76(50.7%) | *0.097* | *0.025* | *0.938* |

T:Total, M:Male, F:Female, NW: normal weight, OW: overweight, *T: p value derived from total of OW group compared with NW group, ^#^M: p value derived from males in the OW group compared with NW group, ^F: p value derived from females in the OW group compared with NW group

- 1. Normal weight (BMI 18.5-24.99) Vs Generalized obesity (GO)

| Disease category | NW(BMI 18.5-24.9) | | | GO (BMI≥30) | | | P values | | |
| --- | --- | --- | --- | --- | --- | --- | --- | --- | --- |
|  | T | M | F | T | M | F | *T | ^#^M | ^F |
| Dyslipidemia | 129(17%) | 55(42.6%) | 74(57.4%) | 43(27.6%) | 10(23.3%) | 33(76.7%) | 0.002 | 0.215 | 0.049 |
| Heart failure | 41(5.4%) | 31(75.6%) | 10(24.4%) | 10(6.4%) | 4(40%) | 6(60%) | 0.622 | 0.921 | 0.190 |
| Hypertension | 243(32.1%) | 121(49.8%) | 122(50.2%) | 92(59%) | 30(32.6%) | 62(67.4%) | <0.0001 | <0.0001 | <0.0001 |
| Ischemic heart disease | 137(18.1%) | 82(59.9%) | 55(40.1%) | 37(23.7%) | 16(43.2%) | 21(56.8%) | 0.104 | 0.054 | 0.388 |
| Osteoarthritis | 9(1.2%) | 3(33.3%) | 6(66.7%) | 7(4.5%) | - | 7(100%) | 0.004 | 0.541 | 0.01 |
| Type 2 diabetes mellitus | 283(37.4%) | 142(50.2%) | 141(49.8%) | 77(49.4%) | 26(33.8%) | 51(66.2%) | 0.005 | 0.019 | 0.230 |

T:Total, M:Male, F:Female, NW: normal weight, GO: generalized obesity, *T: p value derived from total of GO group compared with NW group, ^#^M: p value derived from males in the GO group compared with NW group, ^F: p value derived from females in the GO group compared with NW group

- 1. Low risk WC (<102 in M, <88 in F) Vs Central obesity (WC≥102 in M, ≥88 in F)

| Disease category | Low risk [WC<102(M), <88(F)] | | | CO[WC≥102(M), ≥88(F)] | | | P values | | |
| --- | --- | --- | --- | --- | --- | --- | --- | --- | --- |
|  | T | M | F | T | M | F | *T | ^#^M | ^F |
| Dyslipidemia | 51(11.4) | 18(35.3%) | 33(64.7%) | 34(19.2%) | 2(5.9%) | 32(94.1%) | 0.01 | 0.751 | 0.435 |
| Heart failure | 36(8%) | 27(75%) | 9(25%) | 12(6.7%) | 1(8.3%) | 11(91.7%) | 0.576 | 0.328 | 0.357 |
| Hypertension | 194(43.2%) | 105(54.1%) | 89(45.9%) | 117(65.7%) | 15(12.8%) | 102(87.2%) | <0.0001 | 0.036 | <0.0001 |
| Ischemic heart disease | 96(21.4%) | 52(54.2%) | 44(45.8%) | 52(29.1%) | 8(15.4%) | 44(84.6%) | 0.041 | 0.126 | 0.280 |
| Osteoarthritis | 8(1.8%) | 2(25%) | 6(75%) | 7(3.9%) | - | 7(100%) | 0.115 | 0.66 | 0.516 |
| Type 2 diabetes mellitus | 177(39.4%) | 97(54.8%) | 80(45.2%) | 111(62.4%) | 16(14.4%) | 95(85.6%) | <0.0001 | 0.005 | <0.0001 |

T:Total, M:Male, F:Female, CO: Central obesity, *T: p value derived from total of CO group compared with low risk group, ^#^M: p value derived from males in the CO group compared with low risk group, ^F: p value derived from females in the CO group compared with low risk group

- 1. Normal BMI/WC Vs generalized/central obesity groups

| Disease category | Normal BMI(18.5-24.9)/Normal WC [WC<102(M), <88(F)]  N=1206 | | | GO (BMI≥30)/ CO[WC≥102(M), ≥88(F)]  N=335 | | | P values | | |
| --- | --- | --- | --- | --- | --- | --- | --- | --- | --- |
|  | T | M | F | T | M | F | *T | ^#^M | ^F |
| Dyslipidemia | 180(14.9%) | 73(40.6%) | 107(59.4%) | 77(23.1%) | 12(15.6%) | 65(84.4%) | <0.0001 | 0.165 | 0.137 |
| Heart failure | 77(6.4%) | 58(75.3%) | 19(24.7%) | 22(6.6%) | 5(22.7%) | 17(77.3%) | 0.904 | 0.569 | 0.067 |
| Hypertension | 437(36.2%) | 226(51.7%) | 211(48.3%) | 209(62.6%) | 45(21.5%) | 164(78.5%) | <0.0001 | <0.0001 | <0.0001 |
| Ischemic heart disease | 233(19.3%) | 134(57.5%) | 99(42.5%) | 89(26.6%) | 24(27%) | 65(73%) | 0.004 | 0.015 | 0.048 |
| Osteoarthritis | 17(1.4%) | 5(29.4%) | 12(70.6%) | 14(4.2%) | - | 14(100%) | 0.001 | 0.454 | 0.023 |
| Type 2 diabetes mellitus | 460(38.1%) | 239(52%) | 221(48%) | 188(56.3%) | 42(22.3%) | 146(77.7%) | <0.0001 | <0.0001 | <0.0001 |

T:Total, M:Male, F:Female, GO: Generalized obesity, CO: central obesity, *T: p value derived from total of Generalized/central obesity group compared with Normal BMI/WC group, ^#^M: p value derived from males in the Generalized/central obesity group compared with Normal BMI/WC group, ^F: p value derived from females in the Generalized/central obesity group compared with Normal BMI/WC group

1. Table 10: Chronic diseases prevalence in ‘high risk’ body mass index groups and waist circumference groups in comparison to ‘normal’ in Asian cut-offs.
   1. Normal weight (BMI 18.5-23) Vs overweight/ Increased risk (BMI 23-27.5)

| Disease category | NW(BMI 18.5-23)  N=511 | | | OW(BMI 23-27.5)  N=467 | | | P values | | |
| --- | --- | --- | --- | --- | --- | --- | --- | --- | --- |
|  | T | M | F | T | M | F | *T | ^#^M | ^F |
| Dyslipidemia | 84(16.4%) | 37(44%) | 47(56%) | 88(18.8%) | 32(36.4%) | 56(63.6%) | 0.324 | 0.964 | 0.286 |
| Heart failure | 29(5.7%) | 25(86.2%) | 4(13.8%) | 29(6.2%) | 20(69%) | 9(31%) | 0.724 | 0.813 | 0.154 |
| Hypertension | 163(31.9%) | 84(51.5%) | 79(48.5%) | 172(36.8%) | 85(49.4%) | 87(50.6%) | 0.104 | 0.190 | 0.388 |
| Ischemic heart disease | 90(17.6%) | 55(61.1%) | 35(38.9%) | 102(2.18%) | 64(62.7%) | 38(37.3%) | 0.096 | 0.058 | 0.669 |
| Osteoarthritis | 5(1%) | 1(20%) | 4(80%) | 10(2.1%) | 2(20%) | 8(80%) | 0.141 | 0.476 | 0.238 |
| Type 2 diabetes mellitus | 191(37.4%) | 96(50.3%) | 95(49.7%) | 186(39.8%) | 92(49.5%) | 94(50.5%) | 0.431 | 0.339 | 0.990 |

T:Total, M:Male, F:Female, NW: normal weight, OW: over weight, *T: p value derived from total of OW group compared with NW group, ^#^M: p value derived from males in the OW group compared with NW group, ^F: p value derived from females in the OW group compared with NW group

- 1. Normal weight (BMI 18.5-24.99) Vs generalized obesity/Higher high risk

| Disease category | NW(BMI 18.5-23)  N=511 | | | GO(BMI≥27.5)  N=290 | | | P values | | |
| --- | --- | --- | --- | --- | --- | --- | --- | --- | --- |
|  | T | M | F | T | M | F | *T | ^#^M | ^F |
| Dyslipidemia | 84(16.4%) | 37(44%) | 47(56%) | 69(23.8%) | 19(27.5%) | 50(72.5%) | 0.011 | 0.183 | 0.181 |
| Heart failure | 29(5.7%) | 25(86.2%) | 4(13.8%) | 14(4.8%) | 7(50%) | 7(50%) | 0.609 | 0.525 | 0.225 |
| Hypertension | 163(31.9%) | 84(51.5%) | 79(48.5%) | 152(52.4%) | 55(36.2%) | 97(63.8%) | <0.0001 | <0.0001 | <0.0001 |
| Ischemic heart disease | 90(17.6%) | 55(61.1%) | 35(38.9%) | 65(22.4%) | 32(49.2%) | 33(50.8%) | 0.098 | 0.015 | 0.602 |
| Osteoarthritis | 5(1%) | 1(20%) | 4(80%) | 11(3.8%) | - | 11(100%) | 0.006 | 0.545 | 0.029 |
| Type 2 diabetes mellitus | 191(37.4%) | 96(50.3%) | 95(49.7%) | 134(46.2%) | 55(41%) | 79(59%) | 0.014 | 0.001 | 0.929 |

T:Total, M:Male, F:Female, NW: normal weight, GO: generalized obesity, *T: p value derived from total of GO group compared with NW group, ^#^M: p value derived from males in the GO group compared with NW group, ^F: p value derived from females in the GO group compared with NW group

- 1. Low risk WC (<90 in M, <80 in F) Vs Central obesity (WC≥90 in M, ≥80 in F)

| Disease category | Low risk [WC<90(M), <80(F)] | | | CO[WC≥90(M), ≥80(F)] | | | P values | | |
| --- | --- | --- | --- | --- | --- | --- | --- | --- | --- |
|  | T | M | F | T | M | F | *T | ^#^M | ^F |
| Dyslipidemia | 38(11.6%) | 12(31.6%) | 26(68.4%) | 47(15.8%) | 8(17%) | 39(83%) | 0.119 | *0.277* | *0.659* |
| Heart failure | 28(8.5%) | 20(71.4%) | 8(28.6%) | 20(6.7%) | 8(40%) | 12(60%) | 0.391 | *0.902* | *0.812* |
| Hypertension | 138(41.9%) | 77(55.8%) | 61(44.2%) | 173(58.1%) | 43(24.9%) | 130(75.1%) | <0.0001 | *0.048* | *0.015* |
| Ischemic heart disease | 66(20.1%) | 39(59.1%) | 27(40.9%) | 82(27.4%) | 21(25.6%) | 61(74.4%) | 0.03 | *0.3* | *0.126* |
| Osteoarthritis | 4(1.2%) | 2(50%) | 2(50%) | 11(3.7%) | - | 11(100%) | 0.043 | *0.358* | *0.093* |
| Type 2 diabetes mellitus | 119(36.2%) | 62(52.1%) | 57(47.9%) | 169(56.7%) | 51(30.2%) | 118(69.8%) | <0.0001 | *<0.0001* | *0.049* |

T:Total, M:Male, F:Female, CO: Central obesity, *T: p value derived from total of CO group compared with low risk group, ^#^M: p value derived from males in the CO group compared with low risk group, ^F: p value derived from females in the CO group compared with low risk group

- 1. Chronic diseases which are associated with obesity in normal BMI/WC Vs generalized/central obesity groups

| Disease category | Normal BMI(18.5-23)/Normal WC [WC<90(M), <80(F)] | | | GO (BMI≥27.5)/ CO[WC≥90(M), ≥80(F)] | | | P values | | |
| --- | --- | --- | --- | --- | --- | --- | --- | --- | --- |
|  | T | M | F | T | M | F | *T | ^#^M | ^F |
| Dyslipidemia | 122(14.5%) | 49(40.2%) | 73(59.8%) | 116(19.8%) | 27(23.3%) | 89(76.7%) | 0.009 | 0.108 | 0.647 |
| Heart failure | 57(6.8%) | 45(78.9%) | 12(21.1%) | 34(5.8%) | 15(44.1%) | 19(55.9%) | 0.44 | 0.605 | 0.369 |
| Hypertension | 301(35.8%) | 161(53.5%) | 140(46.5%) | 325(55.3%) | 98(30.2%) | 227(69.8%) | <0.0001 | <0.0001 | <0.0001 |
| Ischemic heart disease | 156(18.6%) | 94(60.3%) | 62(39.7%) | 147(25%) | 53(36.1%) | 94(63.9%) | 0.004 | 0.012 | 0.054 |
| Osteoarthritis | 9(1.1%) | 3(33.3%) | 6(66.7%) | 22(3.7%) | - | 22(100%) | 0.001 | 0.280 | 0.007 |
| Type 2 diabetes mellitus | 310(36.9%) | 158(50.9%) | 152(49%) | 303(51.5%) | 106(35%) | 197(65%) | <0.0001 | <0.0001 | 0.112 |

T:Total, M:Male, F:Female, GO: Generalized obesity, CO: central obesity, *T: p value derived from total of Generalized/central obesity group compared with Normal BMI/WC group, ^#^M: p value derived from males in the Generalized/central obesity group compared with Normal BMI/WC group, ^F: p value derived from females in the Generalized/central obesity group compared with Normal BMI/WC group

1. Table 11: Chronic diseases prevalence in underweight in comparison to ‘normal’
   1. Normal weight (BMI 18.5-24.9) Vs Underweight (BMI <18.5); International cut-offs

| Disease category | NW(BMI 18.5-24.9) | | | UW (BMI <18.5) | | | P values | | |
| --- | --- | --- | --- | --- | --- | --- | --- | --- | --- |
|  | T | M | F | T | M | F | *T | ^#^M | ^F |
| Dyslipidemia | 129(17%) | 55(42.6%) | 74(57.4%) | 16(7%) | 7(43.8%) | 9(56.3%) | *<0.0001* | *0.023* | *0.002* |
| Heart failure | 41(5.4%) | 31(75.6%) | 10(24.4%) | 11(4.8%) | 1(9.1%) | 10(90.9%) | *0.706* | *0.007* | *0.006* |
| Hypertension | 243(32.1%) | 121(49.8%) | 122(50.2%) | 50(21.7%) | 28(56%) | 22(44%) | *0.003* | *0.190* | *0.002* |
| Ischemic heart disease | 137(18.1%) | 82(59.9%) | 55(40.1%) | 31(13.5%) | 16(51.6%) | 15(48.4%) | *0.103* | *0.1* | *0.562* |
| Osteoarthritis | 9(1.2%) | 3(33.3%) | 6(66.7%) | - | - | - | *0.097* | *0.348* | *0.163* |
| Type 2 diabetes mellitus | 283(37.4%) | 142(50.2%) | 141(49.8%) | 37(16.1%) | 19(51.4%) | 18(48.6% | *<0.0001* | *<0.0001* | *<0.0001* |

T:Total, M:Male, F:Female, NW: normal weight, UW: under weight, *T: p value derived from total of UW group compared with NW group, ^#^M: p value derived from males in the UW group compared with NW group, ^F: p value derived from females in the UW group compared with NW group

- 1. Normal weight (BMI 18.5-23) Vs Underweight (BMI <18.5); Asian cut-offs

| Disease category | NW(BMI 18.5-23)  N=511 | | | UW (BMI <18.5)  N=232 | | | P values | | |
| --- | --- | --- | --- | --- | --- | --- | --- | --- | --- |
|  | T | M | F | T | M | F | *T | ^#^M | ^F |
| Dyslipidemia | 84(16.4%) | 37(44%) | 47(56%) | 16(6.9%) | 7(43.8%) | 9(56.3%) | <0.0001 | 0.031 | 0.003 |
| Heart failure | 29(5.7%) | 25(86.2%) | 4(13.8%) | 11(4.7%) | 1(9.1%) | 10(90.9%) | 0.601 | 0.003 | 0.002 |
| Hypertension | 163(31.9%) | 84(51.5%) | 79(48.5%) | 50(21.6%) | 28(56%) | 22(44%) | 0.004 | 0.179 | 0.004 |
| Ischemic heart disease | 90(17.6%) | 55(61.1%) | 35(38.9%) | 31(13.4%) | 16(51.6%) | 15(48.4%) | 0.146 | 0.133 | 0.644 |
| Osteoarthritis | 5(1%) | 1(20%) | 4(80%) | - | - | - | 0.13 | 0.513 | 0.159 |
| Type 2 diabetes mellitus | 191(37.4%) | 96(50.3%) | 95(49.7%) | 37(15.9%) | 19(51.4%) | 18(48.6%) | <0.0001 | <0.0001 | <0.0001 |

T:Total, M:Male, F:Female, NW: normal weight, UW: underweight, *T: p value derived from total of UW group compared with NW group, ^#^M: p value derived from males in the UW group compared with NW group, ^F: p value derived from females in the UW group compared with NW group

Supplementary figures


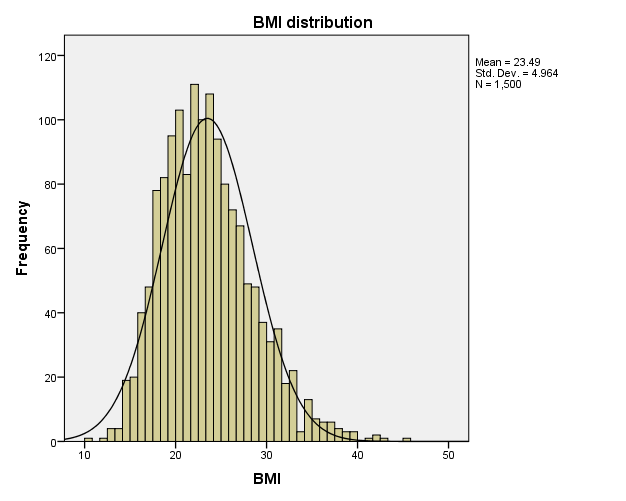


Figure 1: body mass index (a) and waist circumference (b) distribution of the total hospital cohort

(a)
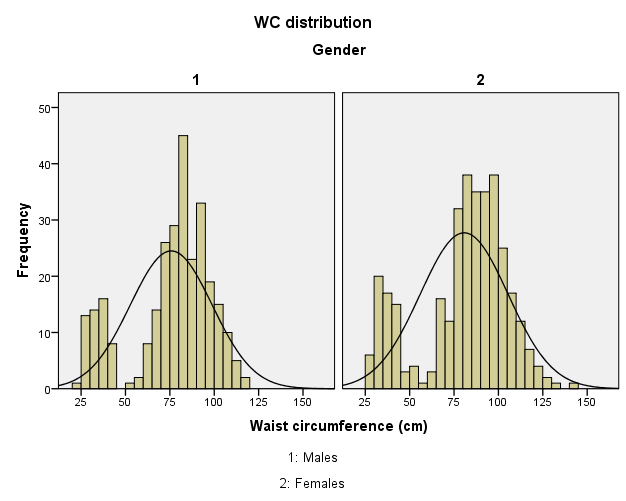
(b)

Figure 1: body mass index (a) and waist circumference (b) distribution of the total hospital cohort
