# Supplementary material for: Influence of Overweight and Obesity on Morbidity and Mortality among Hospitalized Patients in Sri Lanka: A Single-Center Analysis
Source: J Obes. 2022 Aug 18;2022:9172365. doi: 10.1155/2022/9172365 (PMC9411002; doi:10.1155/2022/9172365)
Supplement: Supplementary Materials — Supplementary File 1: tables. Supplementary File 2: questionnaire. Supplementary File 3: STROBE statement—checklist of items that should be included in reports of cohort studies. [file 9172365.f1.zip › 9172365.f1/Supplementary file 2 (2).docx]

**Supplementary file 2:**

**Title: Burden of overweight and obesity and its relation to mortality and morbidity among patients admitting to general medical wards in National hospital of Sri lanka: a prospective observational study**

**Data collection form**

Serial Number……… BHT Number………... Date of collection of Information…………..

**Demographic details**

1. Patients Full Name………………………………………………………………………….
2. Age……………. 3. Sex………. 4. Ethnicity…………………………….

**Anthropometric parameters on admission**

1. Height……… 2. Weight……………. 3. Waist circumference: …………...

**Hospital stay**

1. Date of admission:………………………..
2. Date of discharge/Death:…………………………
3. What is the cause of death if the patient died:…………………………
4. ICU care given ☐Yes ☐No If yes, number of days……………
5. Number of hospital admissions during previous year : ………………………

**Medical condition**

1. Diagnosis/ active medical conditions of the current hospital admission:

…………………………………………………………………………..

…………………………………………………………………………..

………………………………………………………………………….

1. Co-morbidities:

| **Medical condition** | **Diagnosed during current admission** | **Diagnosed before the current admission** |
| --- | --- | --- |
| Type 2 diabetes mellitus | ☐Yes ☐No | ☐Yes ☐No |
| Hypertension | ☐Yes ☐No | ☐Yes ☐No |
| Dyslipidaemia | ☐Yes ☐No | ☐Yes ☐No |
| Ischemic heart disease | ☐Yes ☐No | ☐Yes ☐No |
| Heart failure  Clinical:  2D Echo: | ☐Yes ☐No | ☐Yes ☐No |
| Venous thrombosis | ☐Yes ☐No | ☐Yes ☐No |
| Pressure ulcer | ☐Yes ☐No | ☐Yes ☐No |
| Hospital acquired infections (Specify……………..) | ☐Yes ☐No | ☐Yes ☐No |
| OSA | ☐Yes ☐No | ☐Yes ☐No |
| Osteoarthritis | ☐Yes ☐No | ☐Yes ☐No |
